# A Combination of Ultrasound Characteristics with Macroscopic and Biochemical Features to Diagnose the Etiology of Lymphocytic Pleural Effusions

**DOI:** 10.3390/diagnostics13233502

**Published:** 2023-11-22

**Authors:** Nevenka Piskac Zivkovic, Danijel Cvetko, Marcela Zivkovic, Brankica Simac, Marina Zelenika Margeta, Ivona Kovacevic, Linda Malnar Janes, Kristina Lalic, Zvonka Guzvinec, Anja Ljilja Posavec, Hrvoje Feljan, Mirna Vergles, Zeljko Kuster

**Affiliations:** 1 Special Hospital Radiochirurgia Zageb, 10000 Zagreb, Croatia; hrvoje.feljan@radiochirurgia.hr; 2 Department of Diagnostic and Interventional Radiology, University Hospital Dubrava, 10000 Zagreb, Croatia; dcvetko1@gmail.com; 3 Department of Laboratory Diagnostics, University Hospital Dubrava, 10000 Zagreb, Croatia; mzivkov@kbd.hr (M.Z.); bsimac@kbd.hr (B.S.); 4 Department of Internal Medicine, Division of Pulmonology, University Hospital Dubrava, 10000 Zagreb, Croatia; mzelenika1@kbd.hr (M.Z.M.); ikovacevic@kbd.hr (I.K.); lmalnar@kbd.hr (L.M.J.); klalic@kbd.hr (K.L.); mkorica@kbd.hr (M.V.); 5 Department of Anesthesiology and ICU, University Hospital Centre Zagreb, 10000 Zagreb, Croatia; zvonka184@gmail.com; 6 Polyclinic for Respiratory Tract Diseases, 10000 Zagreb, Croatia; anja.ljilja@gmail.com; 7 Department of Ultrasound and Nuclear Medicine, University Hospital Dubrava, 10000 Zagreb, Croatia; intelus.hr@gmail.com

**Keywords:** lymphocytic pleural effusion, thoracic ultrasound, malignant pleural effusion

## Abstract

Objectives: The primary aim of this study was to improve the diagnosis of lymphocytic pleural effusions (LPEs) by combining their ultrasound characteristics with their macroscopic and biochemical features. Methods: This prospective, single-center, clinical observational study was conducted over a period of three years. The possible malignant etiology of LPEs was assessed using several diagnostic criteria: 1. ultrasound characteristics of the LPEs; 2. typical combinations of macroscopic and ultrasound features; and 3. the logistic regression method with three parameters—pleural nodularity, absence of fibrin, and serum protein concentration. Results: Eighty-four patients with LPEs were included in this study. Pleural nodularity (first criterion) was an ultrasound characteristic that yielded the best individual results (*p* < 0.001) in the differentiation of malignant and nonmalignant etiologies of LPEs (accuracy 73.81%). The combination of the second and third criteria yielded the best results in the prediction of a malignant etiology of LPEs (sensitivity 90.48%, specificity 83.33%, PPV 84.44%, NPV 89.74%, accuracy 86.90%). Based on the results of this prospective study, a protocol for the diagnostic procedure of lymphocytic pleural effusions without a definitive fluid diagnosis has been proposed. Conclusions: A combination of the ultrasound characteristics of LPEs and their macroscopic and biochemical features has improved the predictive accuracy for the malignant etiology of LPEs.

## 1. Introduction

Pleural effusion results from an accumulation of fluid in the pleural space. In lymphocytic pleural effusion, the lymphocyte count exceeds 50% of the cellular composition. More than half of malignant pleural effusions (MPEs) are of this type of pleural effusion [1]. In the differential diagnosis, tuberculosis (especially if the lymphocyte count is >80%), sarcoidosis, post-pericardiotomy state, rheumatoid arthritis (RA), and other systemic autoimmune diseases have to be considered [2,3]. Distinguishing malignancy from specific lymphocytic pleural effusions presents a clinical problem in cases with negative microbiological and cytological findings [4]. Due to its high diagnostic accuracy, pleural biopsy is the gold standard for confirming the malignant etiology of pleural effusion, especially in the case of radiologically suspected malignant mesothelioma. According to clinical guidelines, invasive diagnostic methods, medical thoracoscopy, or video-assisted thoracic surgery (VATS) should be used when pleural fluid analysis and needle biopsy guided by ultrasound or CT are non-diagnostic [5]. In 75% of cases, malignant pleural effusions result from metastatic lung or breast tumors, or lymphomas [6].

Tuberculous pleuritis is the most common form of extrapulmonary tuberculosis in Croatia [7]. Cytological analysis of the pleural effusion showed a lymphocytic predominance in 60 to 90% of the patients. The gold standard for the diagnosis of tuberculous pleuritis is the isolation of *Mycobacterium tuberculosis* from pleural effusion or from pleural biopsy material. Due to the low sensitivity of this diagnostic method (<30%), further invasive pulmonology workup is indicated, primarily pleural biopsy (sensitivity, 97 to 100%) [7,8,9]. In regions with a high incidence of tuberculosis, adenosine deaminase (ADA) analysis of pleural effusion may be used to confirm or rule out tuberculous pleuritis [10]. In regions with a moderate or low incidence of tuberculosis, ADA values of >40 U/L in the diagnosis of tuberculous pleuritis had a sensitivity of 97.1%, specificity of 92.9%, PPV of 86.8%, and NPV of 98.5%. False-positive findings have been reported in cases of pleural empyema and malignant pleural effusion [11].

Multislice spiral computed tomography (MSCT) of the thorax is a mandatory diagnostic method for suspected malignant pleural effusion [12,13,14]. Thoracic ultrasound is an important diagnostic method for the detection and localization of pleural effusion, as well as for the safe performance of thoracentesis or US-guided needle biopsy. Detailed thoracic ultrasound examinations incorporate the analysis of sonographic features of the effusion, visceral and parietal pleura, and visible lung parenchyma [15,16,17]. Although a definitive diagnosis of malignant effusion is made based on a histological assessment, a thorough analysis of the ultrasound findings has significant diagnostic value. Findings of pleural nodes and pleural thickening greater than 1 cm are indicative for malignan etiology. Bugalho et al. [18] reported these findings in 79% of malignant and only 9% of non-malignant effusions.

The primary aim of this study was to improve the diagnosis of lymphocytic pleural effusion by combining ultrasound characteristics with macroscopic and biochemical features.

## 2. Materials and Methods

### 2.1. Stady Population

This prospective clinical observational study was conducted at a tertiary health facility over three years, after obtaining approval from the Ethics Committee of University Hospital Dubrava, Zagreb. The basic enrollment criteria for participation in the study were as follows: (1) signed informed consent forms, (2) ultrasound findings of pleural effusion in patients aged over 18 years, (3) macroscopic findings of pleural fluid samples from the first thoracentesis, and (4) confirmation of lymphocytic pleural effusion with biochemical characteristics of exudate.

### 2.2. Study Design

In all patients with pleural effusion, thoracic ultrasound was indicated by attending clinicians as a standard diagnostic workup. The study physicians (*n* = 3), blinded to the CT scan results, performed thoracic ultrasound and thoracentesis after signing informed consent forms. During the examinations, consultation was allowed, but further influence on the diagnostic procedures until the final diagnosis was not permitted. After the first thoracentesis, macroscopic findings of the pleural fluid specimens were recorded. Patients were enrolled in the prospective study if they fulfilled the main inclusion criterion, which was confirmation of lymphocytic pleural effusion with biochemical characteristics of exudate. All obtained results (ultrasound characteristics of lymphocytic pleural effusions (LPEs) and their macroscopic and biochemical features) were analyzed after a definitive diagnosis of malignant and non-malignant pleural effusion by standard diagnostic procedures.

### 2.3. Diagnostic Methods

#### 2.3.1. Thoracic Ultrasound

A thorough and systematic bilateral examination of defined thoracic regions was performed anteriorly with the patient in a lying position—regions 1, 2, 3, and 4 on the right and left—followed by an examination of the posterior thorax in the sitting position—regions 5, 6, and 7 on the right and left [19,20]. According to their ultrasound characteristics, pleural effusions were classified as complex septated, homogeneously echogenic, anechogenic, and heterogeneously echogenic or complex non-septated [21]. Additionally, all subjects underwent detailed examinations of the visceral, diaphragmatic, and costal parietal pleura. Pleural thickness was measured and nodularity and pleural adhesions were described. The visually accessible liver parenchyma and peripheral lung parenchyma were also examined. The presence or absence of air bronchograms was recorded in cases with verified peripheral pulmonary infiltrates. The ultrasound system Aloka 7 (Aloka, Ibaraki, Japan) and a convex 2–5 MHz probe were used in the analysis of pleural effusions, visceral and diaphragmatic pleura, and lung and liver parenchyma. A linear 5–10 MHz probe was used to investigate the costal parietal pleura and structures of the thoracic wall. All ultrasound measurements and their interpretation were performed before pleural puncture.

#### 2.3.2. Pleural Puncture and Macroscopic Analysis of Pleural Fluid

The site of pleural puncture was determined after thorough ultrasonographic examinations of the thorax. In cases of bilateral pleural effusion, the site with the greatest amount of fluid was selected for thoracentesis. Further diagnostic procedures included thoracentesis, performed according to the British Thoracic Society (BTS) guidelines [22]. The macroscopic findings of the pleural fluid specimens were recorded first by the study physicians who performed the ultrasound examination, and then before the cytological analysis of the pleural fluid, upon which the samples were referred for standard diagnostic workup (cytology, biochemistry, and microbiology).

Based on their macroscopic appearance, pleural effusions were classified into two categories: serous (clear or opaque) and hemorrhagic (bloody or sanguineous).

#### 2.3.3. The Biochemical and Cytological Analysis of the Pleural Fluid Samples

The biochemical values of each pleural fluid sample were recorded, including total protein concentration (g/L) and lactate dehydrogenase (LDH) activity in the pleural effusion (U/L). To measure the ratio of total protein in the pleural fluid sample and serum, as well as the ratio of LDH activity in the pleural fluid sample and serum, total serum protein (g/L) and serum LDH activity (U/L) were determined for each subject. According to the Light criteria, pleural effusions are categorized as exudates or transudates [23]. Cytological analysis of pleural fluid samples was performed using the standard diagnostic procedure at the Department of Pathology and Cytology. In addition to data on the presence or absence of malignant cells, the percentage of cells found in the pleural effusion (neutrophils, lymphocytes, eosinophils, and mesothelial cells) was recorded for each patient. The diagnostic criterion for lymphocytic pleural effusion was a lymphocyte count of >50% in the cellular composition of the pleural fluid sample.

#### 2.3.4. Procedures for the Confirmation of Malignant and Tuberculous Pleural Effusion

The basic requirement for a diagnosis of malignant pleural effusion is a verified finding of malignant cells in a lymphocytic pleural effusion or pleural biopsy sample. After two subsequent cytological punctures, no malignant cells were found in the pleural effusion and the patient underwent standard pleural biopsy (ultrasound- or CT-guided pleural biopsy) or surgical biopsy (video-assisted thoracoscopic surgery (VATS)). To diagnose tuberculous effusion, one of the following criteria was sufficient: a positive pleural effusion or pleural biopsy findings (Ziehl–Neelsen staining); a positive pleural effusion or pleural biopsy culture (grown on Löwenstein–Jensen or MGIT liquid substrate); adenosine deaminase (ADA) values of >40 U/L in the pleural effusion with a good clinical response in the control period; or granulomatous inflammation with caseous necrosis in a pleural biopsy specimen. The patients were divided into two groups based on the definitive diagnosis: those with malignant pleural effusion and those with non-malignant pleural effusion.

Pleural nodularity is the best ultrasound parameter in the prediction of malignant etiology of lymphocytic pleural effusions (sensitivity, 76.19%;, specificity 71.43%; PPV 72.73%; NPV 75%; accuracy 73.81%). All results were compared with the gold standard according to the results of clinical studies [24].

### 2.4. Statistical Data Analysis

To observe the medium effect (d = 0.6) in the difference of numerical variables between two independent groups with a level of significance of 0.05 and strength of 0.80, the minimum required sample size was 84 respondents (42 per group) (G*Power 3.1.9.4). Data on all measured and observed values were evaluated using univariate statistical analysis (incidence of individual nominal or ordinal variables, their shares, calculation of the statistical parameters of mean tendency, and dispersion). A comparison of the interactions between two measured/observed values was performed using parametric and nonparametric methods (bivariate statistical analysis). Statistical significance was set at *p* = 0.05. All values that yielded statistically significant interdependence were included in the multivariate statistical analysis (logistic regression). The diagnostic values of individual diagnostic procedures or individual diagnostic parameters used in the assessment of malignant pleural effusions were determined by ROC analysis (sensitivity, specificity, positive predictive value, and negative predictive value).

## 3. Results

During the study period, the study physicians performed a thoracic ultrasound on 358 patients, out of whom only 84 patients were accepted and enrolled in the prospective study, with a mean age of 63 years (19–93)—women comprised 35 of the 84 patients (41.5%) and men 58.5%. Enrollment was based on the main inclusion criteria, confirmation of lymphocytic pleural effusion, and biochemical characteristics of the exudate.

Based on the definitive diagnosis of pleural effusion, participants were divided into two groups (Table 1): those with malignant pleural effusion (MPE) and those with nonmalignant lymphocytic pleural effusion (NMPE).

The diagnostic values of individual ultrasound and macroscopic characteristics for the prediction of malignant pleural effusion are shown in Table 2. The ultrasound characteristic (first criterion for the differentiation of malignant and nonmalignant etiologies of lymphocytic pleural effusions) that yielded the best results (*p* < 0.0001) was pleural nodularity. Additionally, statistical significance (*p* < 0.05) was achieved for the following parameters: complex septate and heterogeneous pleural effusion, pleural thickening > 10 mm, and hemorrhagic macroscopic pleural effusion (Table 2).

### 3.1. Typical Combinations of Ultrasound and Macroscopic Characteristics for the Prediction of Nonmalignant Pleural Effusion

The preferential diagnosis of malignant pleural effusion requires the combination of one characteristic of greater sensitivity and another characteristic of greater specificity.

The combination of ultrasound and macroscopic characteristics in the prediction of malignant LPEs (nodularity + nonseptation, nodularity + heteroechogenicity, nodularity + thickening, nodularity + hemorrhagic appearance, nonseptation + thickening, nonseptation + hemorrhagic appearance, heteroechogenicity + thickening, heteroechogenicity + hemorrhagic appearance, thickening + hemorrhagic appearance) achieved very high sensitivity (maximum 97.62%), whereas specificity was low (minimum 26.19%). The diagnostic accuracy of the combined ultrasound and macroscopic characteristics was uniform (range 60.71 to 67.86%). Although combining ultrasound and macroscopic characteristics in the suggested way, with the primary goal of diagnosing malignant pleural effusion, resulted in the selection of a large majority of malignant pleural effusion patients, the advantage of this diagnostic combination is the very careful selection of a group of non-malignant pleural effusion patients, due to the very high negative predictive value (NPV > 80%).

Several combinations of ultrasound and macroscopic characteristics achieved a negative predictive value greater than 90%, among which the combination of the absence of fibrin and hemorrhagic appearance of the pleural effusion (nonseptation + hemorrhagic appearance) was the most selective, yielding a maximum negative predictive value of 93.75%. Consistent with these data, the patient group with macroscopically serous pleural effusion (hemorrhagic appearance = “*N*”) and ultrasound findings of fibrous septation (nonseptation = “*N*”) was a verified patient group with a high likelihood of lymphocytic pleural effusion of nonmalignant etiology (*p* < 0.01). There were further combinations of ultrasound and macroscopic characteristics typical of the diagnosis of non-malignant pleural effusion, as shown in Table 3.

The total number of patients with nonmalignant and malignant pleural effusion is smaller than the total sum of values in the individual columns due to the fact that certain combinations, relating to the same patient, occurred more than once.

### 3.2. Typical Combinations of Ultrasound and Macroscopic Characteristics for the Prediction of Malignant Pleural Effusion

The preferential diagnosis of non-malignant pleural effusion requires the combination of one characteristic of greater specificity and another characteristic of greater sensitivity.

The combination of ultrasound and macroscopic characteristics in the prediction of nonmalignant LPEs (nodularity + nonseptation, nodularity + heteroechogenicity, nodularity + thickening, nodularity + hemorrhagic appearance, nonseptation + thickening, nonseptation + hemorrhagic appearance, heteroechogenicity + thickening, heteroechogenicity + hemorrhagic appearance, thickening + hemorrhagic appearance) achieved adequate specificity (maximum 95.24%), whereas the sensitivity was low (minimum 40.48%). The diagnostic accuracy of combined ultrasound and macroscopic characteristics was uniform (range 65.48 to 78.57%).

Although combining ultrasound and macroscopic characteristics in the suggested way, with the primary goal of diagnosing nonmalignant pleural effusion, resulted in the selection of a large majority of patients with non-malignant pleural effusion, the advantage of this diagnostic combination is the careful selection of a group of patients with malignant pleural effusion, due to the high positive predictive value.

Several combinations of ultrasound and macroscopic characteristics achieved a positive predictive value greater than 80% and were typical of the diagnostic picture of malignant pleural effusion, as shown in Table 4.

### 3.3. Summary of Diagnostic Values of Ultrasound, Macroscopic, and Biochemical Parameters in the Prediction of Pleural Effusion Malignancy

The results of the statistical variance analysis (ANOVA) for biochemical parameters of malignant and nonmalignant lymphocytic pleural effusions (CRP, LDH, pleural effusion, and serum proteins) yielded statistically significant differences in the mean values of the biochemical parameters for serum (s) and pleural fluid (*p*) proteins (*p* < 0.01).

Further multivariate statistical analyses (logistic regression) included all diagnostic indicators that proved relevant (statistically significant) in the individual prediction of pleural effusion malignancy: nodularity, non-septation, thickening, hemorrhagic appearance, proteins (s), proteins (*p*), and heteroechogenicity. Multiple regression analysis showed that the best predictive model for pleural effusion malignancy involved three independent indicators: nodularity, non-septation, and serum protein concentration (s) (Table 5).

### 3.4. Proposal of Diagnostic Procedures for Lymphocytic Pleural Effusions without a Definitive Fluid Diagnosis

**Figure 1:**
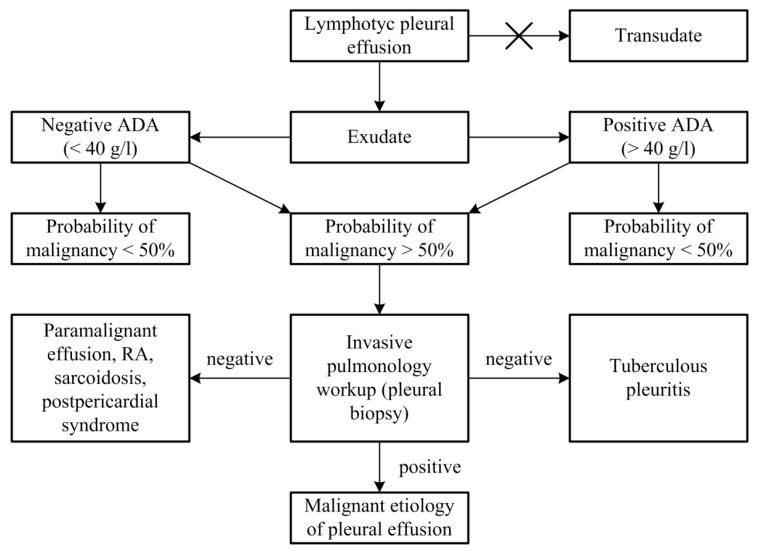
Proposal of diagnostic procedures regarding lymphocytic pleural effusions without definitive fluid diagnosis (no acid-fast bacilli or malignant cells in the fluid). A cross symbol: the pleural effusions categorised as transudates according to the Light criteria were excluded from the study.

Based on the results of this prospective study, a protocol for diagnostic procedures regarding lymphocytic pleural effusions without definitive fluid diagnosis (no acid-fast bacilli or malignant cells in the fluid) was proposed (Figure 1).

Prior to applying the diagnostic procedure protocol for lymphocytic pleural effusions without definitive fluid diagnosis, it is necessary to:

1. Determine ADA levels in the pleural effusion;
2. Perform a repeat chest ultrasound for a detailed pleural and pleural effusion analysis, if not performed before the initial pleural puncture;
3. Estimate the probability of malignant etiology in pleural effusion by applying the second and third criteria.

Diagrams showing the probability of a malignant etiology of lymphocytic pleural effusions based on the application of the third criterion.

**Figure 2:**
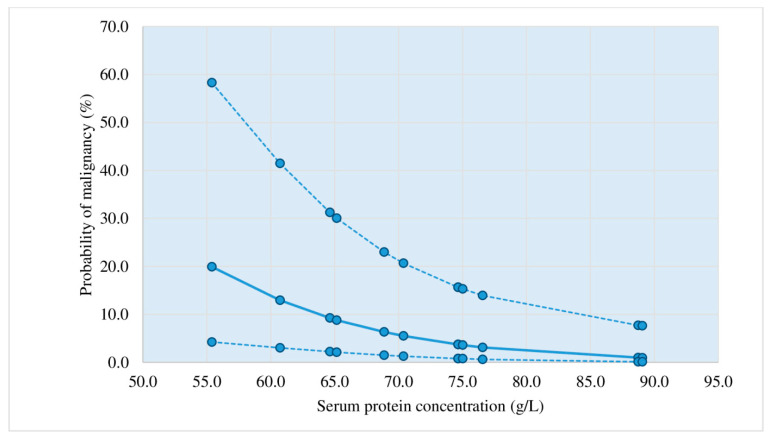
Probability of malignant etiology of pleural effusion estimated by logistic regression (negative nodularity, nonseptation); *n* = 13; dotted line: 95% reliability interval limits.

**Figure 3:**
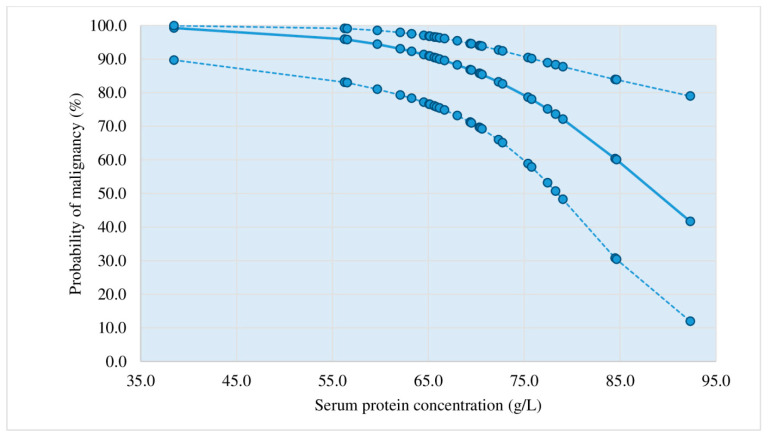
Probability of malignant etiology of pleural effusion estimated by logistic regression (positive nodularity, septation); *n* = 34; dotted line: 95% reliability interval limits. The cut-off value (50%) corresponds with a protein concentration of 88.8 g/L.

**Figure 4:**
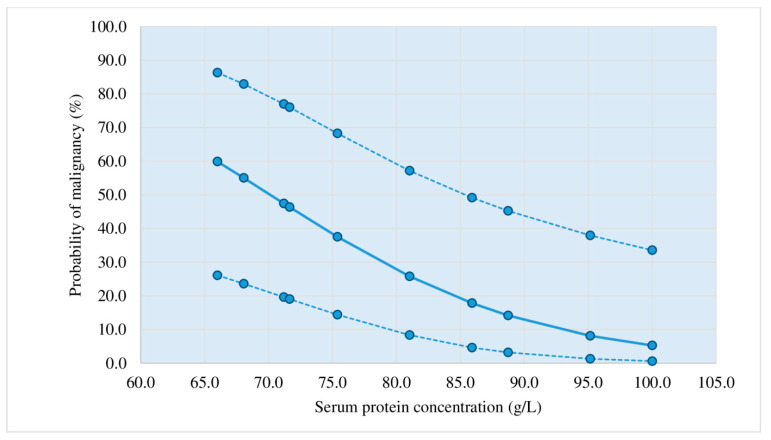
Probability of malignant etiology of pleural effusion estimated by logistic regression (positive nodularity, nonseptation); *n* = 10; dotted line: 95% reliability interval limits. The cut-off value (50%) corresponds with a protein concentration of 70.1 g/L.

**Figure 5:**
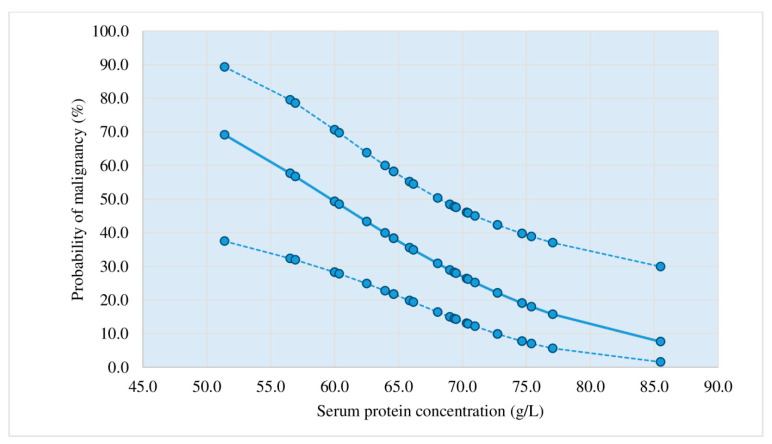
Probability of malignant etiology of pleural effusion estimated by logistic regression (negative nodularity, septation); *n* = 27; dotted line: 95% reliability interval limits. The cut-off value (50%) corresponds with a protein concentration of 59.7 g/L.

Figure 2, Figure 3, Figure 4 and Figure 5 represent diagrams comprising all diagnostic combinations of nodularity and septation, thus enabling the direct association between a specific serum protein concentration and the malignancy probability of the pleural effusion.

Of the 13 patients included in this diagram, 12 had effusions of a nonmalignant etiology. The cutoff value (50%) corresponded to protein concentration of 58 g/L.

## 4. Discussion

The results of this prospective study met our research goals. A combination of the ultrasound characteristics of lymphocytic pleural effusions with their macroscopic and biochemical characteristics improves the accuracy of predicting the malignant etiology of lymphocytic pleural effusions.

The analysis of the ultrasound characteristics (first criterion for the differentiation of malignant and nonmalignant etiology of lymphocytic pleural effusions) yielded a statistically significant difference in the following ultrasound parameters: pleural thickening > 10 mm, pleural nodularity, heterogeneously echogenic, and complex septated or fibrous septated pleural effusion. The best results in the prediction of a malignant etiology of pleural effusion were achieved for pleural nodularity (sensitivity 76.19%; specificity 71.43%; PPV 72.73%; NPV 75%; accuracy 73.81%). A comparison of the ultrasound parameter sensitivity in our study with the data from the study by Bugalho et al. [18] showed that there were no statistical differences between the ultrasound parameters, including the cumulative sensitivity for ultrasound diagnostics. The somewhat lower specificity for pleural nodularity (71.43%) obtained in our study resulted from the fact that there was a significantly higher number of patients with tuberculous lymphocytic pleural effusion in the control group. The total number of tuberculous pleural effusions in the study by Bugalho et al. was 15/67 (22.4%), whereas a specific etiology of the pleural effusion was verified in 29/42 (69%) patients in our study group of nonmalignant pleural effusion patients.

In the results obtained by Bugalho et al. [18], the non-malignant pleural effusion group was not categorized, that is, pleural effusions biochemically characterized as transudates or exudates were included in the same group. The same group of authors reported that the incidence of fibrous septation in nonmalignant pleural effusions showed a statistically significant difference in relation to malignant pleural effusions (*p* = 0.006), mostly in cases of parapneumonic and tuberculous pleural effusions. The absence of fibrous septation in malignant pleural effusions had a sensitivity of 92.4%, a specificity of 25.4%, a PPV of 54.9%, and an NPV of 77.3%. However, the statistical significance of the absence of fibrous septation within the pleural effusion as a predictor of malignant etiology (*p* = 0.585) was not confirmed by logistic regression analysis.

Our study group with nonmalignant pleural effusions was categorized (exudates with lymphocytic predominance). The absence of fibrous septation as a predictor of the malignant etiology of a lymphocytic pleural effusion showed a sensitivity of 90.48% and a specificity of 45.24% (PPV 62.30%, NPV 82.61%).

Chen et al. [15] analyzed malignant and specific/tuberculous lymphocytic pleural effusions. They reported fibrous septation in 47% of specific and 4% of malignant pleural effusions, thus concluding that fibrous septation in lymphocytic pleural effusions was a predictor of their specific etiology in regions with a high incidence of tuberculosis (sensitivity 47%, specificity 96%, PPV 94%, NPV 59%). Our results show a 9.52% incidence of fibrous septation in malignant pleural effusions. The somewhat lower sensitivity (45.24%) and specificity (90.48%) obtained in our study group resulted from the nonmalignant lymphocytic but nonspecific pleural effusions (RA, postpericardiotomy state, sarcoidosis), which showed fibrous septation in a smaller percentage (23.07%), as opposed to tuberculous pleuritis (55.17%).

However, if the macroscopic and biochemical characteristics of pleural effusions are analyzed in addition to their ultrasound characteristics, the predictive results for the malignant etiology of lymphocytic pleural effusions are significantly different.

Pleural nodularity was the best individual ultrasound parameter in the prediction of a malignant etiology of lymphocytic pleural effusions (sensitivity 76.19%, specificity 71.43%, PPV 72.73%, NPV 75%, accuracy 73.81%), according to results of other clinical studies [24]. Several combinations of ultrasound and macroscopic characteristics for the prediction of malignant pleural effusion were singled out by bivariate statistical analysis: (1) pleural nodularity and macroscopically hemorrhagic/sanguineous pleural effusion; (2) pleural nodularity and absence of fibrin; (3) pleural thickening > 10 mm and hemorrhagic/sanguineous pleural effusion; and (4) heteroechogenic ultrasound findings in a macroscopically hemorrhagic/sanguineous pleural effusion (Table 4).

The same statistical method was used to identify several combinations of ultrasound and macroscopic characteristics for the prediction of non-malignant pleural effusion: (1) non-hemorrhagic and fibrous septated pleural effusion; (2) fibrous septated pleural effusion without pleural nodularity; (3) fibrous septated pleural effusion without pleural thickening > 10 mm; and (4) pleural effusion with neither heteroechogenic ultrasound characteristics nor pleural nodularity (Table 3).

Better results in the prediction of the malignant etiology of lymphocytic pleural effusions (Table 5) were obtained when typical combinations of ultrasound and macroscopic characteristics of malignant and non-malignant pleural effusions (criterion 2) were used in comparison with pleural nodularity (criterion 1). However, as 10 patients (12%) did not present with the typical combination of ultrasound and macroscopic characteristics of malignant or non-malignant lymphocytic pleural effusions, this method is not fully applicable to all patients. The third criterion was also used to predict the malignant etiology of lymphocytic pleural effusions.

Logistic regression was applied in the analysis of the ultrasound, macroscopic, and biochemical parameters, which individually showed statistical significance in the differentiation of malignant and nonmalignant lymphocytic pleural effusions: nodularity, nonseptation, thickening, hemorrhagic appearance, proteins (s), proteins (*p*), and heteroechogenicity. The application of this statistical method resulted in three statistically relevant parameters (pleural nodularity, absence of fibrin, and serum protein concentrations) that yielded the largest number of patients with an accurate prediction of malignant and non-malignant etiology of lymphocytic pleural effusions. This diagnostic method (criterion 3) had a sensitivity of 73.81%, a specificity of 83.33%, a PPV of 81.58%, an NPV of 76.09%, and an accuracy of 78.57%; in addition, it showed improvement in relation to the first criterion of pleural nodularity (Table 5, Figure 2, Figure 3, Figure 4 and Figure 5). Samanta S et al. [25] reported significantly higher serum protein concentrations in the tuberculosis group than in the lung cancer group (*p*-value < 0.0001). In the ROC curve for TB vs. lung cancer, the best cut-off value for serum proteins was 6.2 gm/dL (sensitivity 92.0%, specificity 100%). The best results in the prediction of malignant etiology of pleural effusions were achieved by the combined application of the second criterion, a fast and simple method, and in patients presenting without the typical combination of ultrasound and macroscopic characteristics, the third criterion (logistic regression method), with a sensitivity of 90.48%, a specificity of 83.33%, a PPV of 84.44%, an NPV of 89.74%, and an accuracy of 86.90%.

Based on the results of this prospective study, a protocol for diagnostic procedures for lymphocytic pleural effusions without definitive fluid diagnosis (no acid-fast bacilli or malignant cells in the fluid) was proposed (Figure 1). Before applying the protocol, we must 1. determine ADA levels in pleural effusion, 2. perform a repeat chest ultrasound for a detailed pleural and pleural effusion analysis, if this was not performed before the initial pleural puncture, and 3. estimate the probability of a malignant etiology of pleural effusion by applying the second and third criteria. Patients who showed a typical combination of ultrasound and macroscopic characteristics of malignant pleural effusion (nodularity/hemorrhagic effusion, nodularity/nonseptation, hemorrhagic effusion/thickening > 10 mm, heteroechogenicity/hemorrhagic effusion) (Table 4) presented a >50% probability of malignant etiology in the pleural effusion. In cases with typical combinations of nonmalignant effusions (fibrin septation/serous effusion, fibrin septation/no nodularity, fibrin septation/no thickening > 10 mm) (Table 3), the probability of malignant etiology of the pleural effusion was <50%. In patients presenting without the typical combination of malignant or non-malignant pleural effusion, logistic regression for three parameters (pleural nodularity, absence of fibrin in the pleural effusion, and serum protein concentrations) was used to estimate the probability of a malignant etiology of the pleural effusion, as shown in Figure 2, Figure 3, Figure 4 and Figure 5.

The limitations of this prospective clinical study were the lack of a gold standard (pleural biopsies in all patients to compare our results), the relatively small number of participants in both the study and control groups, and the fact that it was conducted at a single site. Data from multiple centers and ultrasound operators in the future will strengthen the validity of our clinical study’s results.

## 5. Conclusions

A combination of the ultrasound characteristics of lymphocytic pleural effusions with their macroscopic and biochemical characteristics will improve the accuracy in predicting the malignant etiology of lymphocytic pleural effusions.

Based on the results of this prospective study, a protocol for diagnostic procedures for lymphocytic pleural effusions without definitive fluid diagnosis (no acid-fast bacilli or malignant cells in the fluid) has been proposed.

## Figures and Tables

**Table 1:** The main groups of patients according to the definitive diagnosis of pleural effusion.

Final Diagnosis Lymphocytic Pleural Effusion		
Malignant Pleural Effusion, MPE (*n*)		42
Lung cancer	Adenocarcinoma	15
	Squamous cell carcinoma	1
	Small cell carcinoma	1
Lymphoma		5
Mesothelioma		3
Breast cancer		6
Gastrointestinal cancer		6
Other malignancies		5
Nonmalignant pleural effusion, NMPE (*n*)		42
Tuberculosis		29
Rheumatoid arthritis		4
Postpericardiotomy syndrome		5
Dressler syndrome		3
Sarcoidosis		1

**Table 2:** Ultrasound and macroscopic characteristics of malignant and nonmalignant pleural effusions.

Ultrasound and Macroscopic Characteristics	MPE (*n* = 42)	NMPE (*n* = 42)	*p*-Value (Significant)	Accuracy
Pleural effusion echogenicity				
Anechogenicity	3	2		
Septation	4	19	*p* < 0.001	0.6786
Homoechogenicity	2	1		
Heteroechogenicity	33	20	*p* < 0.05	0.6190
Parietal and visceral pleura				
Thickening (>10 mm)	31	17	*p* < 0.01	0.6667
Nodularity	32	12	*p* < 0.0001	0.7381
Macroscopic characteristics				
Hemorrhagic appearance	24	11	*p* < 0.01	0.6548
Opacity	13	13		

**Table 3:** Typical combinations of ultrasound and macroscopic characteristics (NPV > 80%) used for the prediction of nonmalignant pleural effusion.

Nonseptation	Heteroechogenicity	Thickening	Nodularity	Hemorrhagic	NMPE (*n*)	MPE (*n*)
*N*				*N*	15	1
*N*			*N*		12	1
*N*		*N*			11	1
	*N*		*N*		18	3
Total (actual) number					30	4

Legend: *n* = number of patients; *N* = negative finding; MPE = malignant pleural effusion; NMPE = non-malignant pleural effusion; hemorrhagic = hemorrhagic appearance.

**Table 4:** Typical combinations of ultrasound and macroscopic characteristics (PPV > 80%) used for the prediction of malignant pleural effusion.

Nonseptation	Heteroechogenicity	Thickening	Nodularity	Hemorrhagic	NMPE (*n*)	MPE (*n*)
			Y	Y	2	21
		Y		Y	4	21
Y			Y		5	29
	Y			Y	4	17
Total (actual) number					7	33

MPE, malignant pleural effusion; NMPE, nonmalignant pleural effusion; *n*, number of patients; Y, positive findings. The total number of patients with nonmalignant and malignant pleural effusion is smaller than the total sum of values in the individual columns due to the fact that certain combinations, relating to the same patient, occurred more than once.

**Table 5:** Summary of diagnostic values of ultrasound, macroscopic, and biochemical parameters in the prediction of pleural effusion malignancy according to established parameters.

Parameter	Sensitivity (%)	Specificity (%)	PPV (%)	NPV (%)	Accuracy (%)
1. criterion (nodularity) ^a^	76.19	71.43	72.73	75.00	73.81
2. criterion (US + macroscopic characteristics) ^b^*	78.57–90.48	71.43–83.33	73.33–84.44	76.92–89.74	75.00–86.90
3. criterion (logistic regression method) ^c^	73.81	83.33	81.58	76.09	78.57

Legend: 1. criterion ^a^—ultrasound characteristics (pleural nodularity as the best single ultrasound parameter for the differentiation of malignant and non-malignant pleural effusion); 2. criterion ^b^—typical combinations of ultrasound and macroscopic characteristics for malignant and nonmalignant pleural effusion, and 3. criterion ^c^—results for prediction of pleural effusion malignancy by logistic regression of three parameters (nodularity, nonseptation, and serum protein concentrations) * For combined ultrasound and macroscopic parameters, due to several patients having undefined status (*n* = 10), the values for sensitivity, specificity, positive and negative predictive values, and accuracy were within the reference range.

## Data Availability

The data presented in this study are available on request from the corresponding author. The data are not publicly available due to data confidentiality.
